# Supplementary material for: Personal and workplace factors influencing the resilience of nurses caring for women with cervical cancer in a resource-constrained setting in Ghana
Source: PLoS One. 2024 Dec 3;19(12):e0314764. doi: 10.1371/journal.pone.0314764 (PMC11614206; doi:10.1371/journal.pone.0314764)
Supplement: S1 Appendix — (DOCX) [file pone.0314764.s001.docx]

**APPENDIX 1
INTERVIEW GUIDE**

***Preamble***

*Thank you for agreeing to participate in this study. The purpose of this study is to explore resilience among nurses and midwives caring for advanced-stage cervical cancer. Resilience simply means how you are able to cope with stressful events and gain positive experience from them. The specific objectives are to identify individual characteristics of nurses and midwives caring for cervical cancer patients and how they affect resilience, explore workplace characteristics which affect resilience, describe challenges encountered by nurses and midwives caring for advanced-stage cervical cancer patients and to explore available support systems for nurses and midwives caring for advanced stage cervical cancer patients. If any questions are not clear to you, feel free to ask me for clarification. If there is any question you are not comfortable answering, you are very free not to answer. Remember, participation is entirely voluntary.*

Date: ________________ Code no: ____________

**Section 1: Backgrounf Information**

Age (In Years): 21-30 ☐ 31-40 ☐ 41-50 ☐ 51-60 ☐

Educational Level: Diploma ☐ BSc ☐ Masters ☐ Ph.D. ☐

Religion: Christian ☐ Islam ☐ Other ☐

Marital Status: Single ☐ Married ☐ Separated ☐ Divorced ☐ Co-habitation ☐

No of children if any: One ☐ Two ☐ Three ☐ Four and more ☐

No of dependents: One ☐ Two ☐ Three ☐ Four and more ☐

Years of experience as nurse/ midwife:

Less than three ☐ three to five ☐ six to ten ☐ Above ten years ☐

**Section 2: How Personal characteristics influence resilience**

1. How does a person become resilient?
2. How does your educational level influence your ability to cope with the stresses in your work?
3. Share with me how you combine marriage life with work life
4. Describe how your

**Section 3: Workplace characteristics which affect resilience**

1. What factors in your work environment causes stress for you?
2. Describe the last time you were extremely stressed at work
3. Why were you stressed?
4. How did you manage the stress?
5. Could you avoid such stress at your workplace in future?
6. Share with me how the factors listed above influences your ability to cope with the stresses in your work?

**Section 4: Challenges encountered by nurses and midwives caring for advanced stage cervical cancer patients and to**

1. Tell me about challenges you encounter in your work
2. What has been your greatest failure in your course of work
3. Do you think this has shaped you as a person?
4. What will you do differently next time?
5. Share with me how your workplace challenges have affected your personal life
6. Tell me about any health challenges you have faced as a result of your work

**Section 5: Support systems for nurses and midwives caring for advanced stage cervical cancer patients**

1. Tell me about support systems that help you cope
2. Tell me about the support systems at your workplace
3. Discuss with me how you personally deal with stress
4. How do you keep your fellow co-workers upbeat at the workplace
5. Share with me how you think resilience can be developed
