# Supplementary material for: Personal and workplace factors influencing the resilience of nurses caring for women with cervical cancer in a resource-constrained setting in Ghana
Source: PLoS One. 2024 Dec 3;19(12):e0314764. doi: 10.1371/journal.pone.0314764 (PMC11614206; doi:10.1371/journal.pone.0314764)
Supplement: Attachment — Submitted filename: Response to Reviewers.pdf [file pone.0314764.s002.pdf]

University of Ghana
College of Health Sciences
School of Nursing and Midwifery
Department of Adult Health

1st October, 2024

The Editor
PLOS ONE

Dear Sir/Madam,

RESPONSE TO REVIEWERS

We are grateful for the review of our manuscript titled "**Personal and workplace factors influencing the resilience of nurses caring for women with cervical cancer in resource constained setting in Ghana**" to make it better for possible publication.

Comments from the editor
Journal Requirements:
1. When submitting your revision, we need you to address these additional requirements.

Please ensure that your manuscript meets PLOS ONE's style requirements, including those for file naming. The PLOS ONE style templates can be found at https://journals.plos.org/plosone/s/file?id=wjVg/PLOSOne_formatting_sample_main_body.pdf and https://journals.plos.org/plosone/s/file?id=ba62/PLOSOne_formatting_sample_title_authors_affiliations.pdf

2. Please provide a complete Data Availability Statement in the submission form, ensuring you include all necessary access information or a reason for why you are unable to make your data freely accessible. If your research concerns only data provided within your submission, please write "All data are in the manuscript and/or supporting information files" as your Data Availability Statement.

3. PLOS requires an ORCID iD for the corresponding author in Editorial Manager on papers submitted after December 6th, 2016. Please ensure that you have an ORCID iD and that it is validated in Editorial Manager. To do this, go to 'Update my Information' (in the upper left-hand corner of the main menu), and click on the Fetch/Validate link next to the ORCID field. This will take you to the ORCID site and allow you to create a new iD or authenticate a pre-existing iD in Editorial Manager.

4. In the online submission form, you indicated that "Data is available upon request from the corresponding author".

All PLOS journals now require all data underlying the findings described in their manuscript to be freely available to other researchers, either 1. In a public repository, 2. Within the manuscript itself, or 3. Uploaded as supplementary information.
This policy applies to all data except where public deposition would breach compliance with the protocol approved by your research ethics board. If your data cannot be made publicly available for ethical or legal reasons (e.g., public availability would compromise patient privacy), please explain your reasons on resubmission and your exemption request will be escalated for approval.

5. Your ethics statement should only appear in the Methods section of your manuscript. If your ethics statement is written in any section besides the Methods, please move it to the Methods section and delete it from any other section. Please ensure that your ethics statement is included in your manuscript, as the ethics statement entered into the online submission form will not be published alongside your manuscript.

6. Please include captions for your Supporting Information files at the end of your manuscript, and update any in-text citations to match accordingly. Please see our Supporting Information guidelines for more information: http://journals.plos.org/plosone/s/supporting-information.
7. Please review your reference list to ensure that it is complete and correct. If you have cited papers that have been retracted, please include the rationale for doing so in the manuscript text, or remove these references and replace them with relevant current references. Any changes to the reference list should be mentioned in the rebuttal letter that accompanies your revised manuscript. If you need to cite a retracted article, indicate the article's retracted status in the References list and also include a citation and full reference for the retraction notice.

[Note: HTML markup is below. Please do not edit.]

Response to editor

1. We have complied with the requirements for file naming as suggested by the editor.
2. We have complied with the suggestions on data availaibility by providing the recommended data availability statement in the manuscript. This is on page 23 of the revised manuscript.
3. The ORCID iD for the corresponding author is already part of the information for the Editorial Manager.
4. We have revised the data availability statements in the submission system as suggested by the editor.
5. We have provided the ethics section in the methods section of the manuscript as suggested by the editor . This is on page 10 of the revised manuscript.
6. We have provided the appropriate captions for the supporting information as suggested by the reviewer and same has been provided at the end of the manuscript.
7. We have reviewed the reference list and ensure they comply with the Journal's specifications.

Reviewers' comments:

Reviewer's Responses to Questions

Comments to the Author

1. Is the manuscript technically sound, and do the data support the conclusions?

The manuscript must describe a technically sound piece of scientific research with data that supports the conclusions. Experiments must have been conducted rigorously, with appropriate controls, replication, and sample sizes. The conclusions must be drawn appropriately based on the data presented.

Reviewer #1: Yes

Reviewer #2: Yes

2. Has the statistical analysis been performed appropriately and rigorously?

Reviewer #1: N/A

Reviewer #2: Yes

3. Have the authors made all data underlying the findings in their manuscript fully available?

The PLOS Data policy requires authors to make all data underlying the findings described in their manuscript fully available without restriction, with rare exception (please refer to the Data Availability Statement in the manuscript PDF file). The data should be provided as part of the manuscript or its supporting information, or deposited to a public repository. For example, in addition to summary statistics, the data points behind means, medians and variance measures should be available. If there are restrictions on publicly sharing data—e.g. participant privacy or use of data from a third party—those must be specified.

Reviewer #1: No

Reviewer #2: Yes

4. Is the manuscript presented in an intelligible fashion and written in standard English?

PLOS ONE does not copyedit accepted manuscripts, so the language in submitted articles must be clear, correct, and unambiguous. Any typographical or grammatical errors should be corrected at revision, so please note any specific errors here.

Reviewer #1: Yes

Reviewer #2: Yes

Review Comments to the Authors

Reviewer #1:

The authors have presented an important research article with the aim of exploring personal and workplace factors influencing the resilience of nurses caring for patients with advanced cervical cancer in a resource constrained setting.

The methodology was well presented however, the authors need to address the following observations:

1. In the data collection section, it was stated that 64 nurses and midwives were contacted but only 48 expressed interests in participating and only 20 participants were interviewed. Were this 20 selected from the 48 after meeting the inclusion criteria?

2. And is 64 the total number of nurses and midwives that work in the unit where the participants were recruited?

3. In the subsection titled 'Rigour', in the first line the authors mention that "the interview guide was pretested among two patients...". Were patients also involved or is this a typographical error?

4. During the interview, were the participants asked the same questions in the same format or did these vary according to participants?

<u>Responses of Authors to Reviewer 1</u>

1. Yes, the twenty participants were selected from the eligible forty-eight participants who were ready to participate in the study. This has been explained on page 6 of the revised manuscript.
2. The 64 were the nurses and midwives at the Unit who met the inclusion criteria and not the total number of nurses working at the unit. This is stated on page 6 of the revised manuscript on data collection.
3. We have rectified the typographical error in the first sentence of the rigour section. The study tool was pre-tested among nurses. This is stated on page 9 of the revised manuscript.
4. We have indicated that the participants were sasked the same questions in the same format with further probing questions to elicit in-depth information. This stated on page 7 of the revised manuscript.

Reviewer #2:

Questions to authors

1. What criteria were used to determine data redundancy and data saturation after the sixteenth interview? And why was it decided to add exactly four respondents?

2. Did authors have criteria for estimation of poor reporting culture of patients?

3. Did authors used participant information sheet in local language ?

<u>Responses of Authors to Reviewer 2</u>

1. The criteria for data redundancy and data saturation has been stated on page 6 of the revised manuscript specifically on the continuation of study population and sample size determination. We have provided our reasons for the addition of the four additional interviews after the 16th interview on page 6 of the revised manuscript.
2. Respectfully, the study was not on patients and we did not collect information on poor reporting culture of patients. This can be explored in future studies.

3. We have indicated that we used participant information sheet in English since all the participants were literate. This has been stated on pages 6 and 10 of the revised manuscript.

Thank you

Yours sincerely,
Dr. Kennedy Dodam Konlan
(Corresponding author)
